# Development and internal validation of prediction models for ICU-level intervention and 14-day mortality at Medical Emergency Team activation: A retrospective cohort study

**DOI:** 10.1371/journal.pone.0360511

**Published:** 2026-10-09

**Authors:** Junji Shimauchi, Takuya Nishino, Kenta Shigeta, Hiroshi Mase, Takeshi Yamamoto, Shoji Yokobori, Masashi Ishikawa

**Affiliations:** 1 Department of Nursing, Nippon Medical School Hospital, Tokyo, Japan; 2 Department of Health Policy and Management, Nippon Medical School, Tokyo, Japan; 3 Department of Emergency and Critical Care Medicine, Nippon Medical School, Tokyo, Japan; 4 Department of Anesthesiology and Pain Medicine, Graduate School of Medicine, Nippon Medical School, Tokyo, Japan; 5 Department of Surgical Intensive Care, Nippon Medical School Hospital, Tokyo, Japan; 6 Division of Cardiovascular Intensive Care, Nippon Medical School Hospital, Tokyo, Japan; Universiti Sains Malaysia, MALAYSIA

## Abstract

**Background:**

Medical Emergency Team (MET) activation represents acute clinical deterioration requiring rapid decisions regarding escalation of care. Previous post-MET prediction studies have evaluated outcomes including unplanned intensive care unit (ICU) admission and mortality; however, ICU admission is a location-based outcome that may be influenced by institutional factors. Prediction based on treatment intensity rather than location of care has received less attention.

**Methods:**

We conducted a retrospective single-center cohort study of adult patients undergoing MET activation on general wards at a tertiary-care hospital in Japan between May 2021 and December 2025. Two outcomes were evaluated: initiation of ICU-level interventions within 24 hours and all-cause 14-day mortality. ICU-level interventions were defined as the initiation of mechanical ventilation, vasopressor or inotropic infusion, renal replacement therapy, or polymyxin B hemoperfusion (PMX). Multivariable logistic regression and Cox proportional hazards models were developed using variables available at the time of MET activation. Model performance was evaluated using discrimination (C-index), calibration, decision curve analysis, and bootstrap-based internal validation.

**Results:**

Among 533 patients, 303 (56.8%) required ICU-level interventions and 147 (27.6%) died within 14 days. Lower albumin levels and higher blood urea nitrogen, potassium, and white blood cell count were associated with both outcomes, whereas higher heart rate was associated with 14-day mortality. The model demonstrated moderate discrimination for ICU-level intervention (C-index 0.721, 95% CI 0.660–0.745) and good discrimination for 14-day mortality (C-index 0.784, 95% CI 0.720–0.814). Calibration analyses indicated modest overfitting but generally good agreement between predicted and observed risks. Decision curve analysis demonstrated clinical net benefit across a range of threshold probabilities.

**Conclusions:**

In this single-center cohort, treatment escalation and short-term mortality represented related but distinct dimensions of clinical risk. Defining intensive care need based on treatment intensity rather than ICU admission may provide an operational framework for risk stratification at MET activation. External validation is required before clinical implementation.

## Introduction

Medical Emergency Teams (MET), also known as Rapid Response Teams, are hospital-based systems designed to identify and manage acute clinical deterioration in ward patients [1, 2]. Since their international dissemination, rapid response systems have become integral components of hospital safety infrastructure [1]. Meta-analyses have reported that their implementation is associated with reductions in in-hospital cardiac arrest and, in some settings, hospital mortality [3, 4]. Contemporary cohort studies further demonstrate that patients triggering MET activation face a substantial risk of subsequent ICU transfer and short-term mortality, highlighting the prognostic significance of these events [5, 6]. MET activation therefore represents a critical clinical inflection point requiring rapid decisions regarding escalation of care, including initiation of organ support and potential ICU transfer. Accurate risk stratification at the time of activation is essential for appropriate triage and allocation of critical care resources.

Abnormal physiological findings at the time of MET activation, including hypotension, tachypnea, hypoxemia, altered mental status, oliguria, and laboratory abnormalities, have been associated with subsequent clinical deterioration and adverse outcomes in hospitalized patients [7, 8]. Previous post-MET prediction studies have evaluated outcomes including unplanned ICU admission and mortality, including the model reported by Batterbury et al. [9]. However, ICU admission is a location-based outcome and may be influenced not only by patient severity but also by bed availability, local triage policies, and clinician judgment [10]. ICU admission thresholds and actual admission rates vary across institutions and are influenced not only by patient severity but also by bed availability, local triage policies, and clinician judgment [11]. Consequently, ICU admission does not necessarily reflect the intensity of critical care delivered. Critically ill patients may receive advanced organ support outside the ICU, whereas some ICU-admitted patients may not require high-intensity support. Prediction based on treatment intensity rather than location of care has received less attention.

The objective of this study was to develop and internally validate prediction models for (1) the initiation of ICU-level interventions within 24 hours after MET activation and (2) 14-day mortality, using clinical variables available at the time of MET activation. By defining intensive care “need” based on treatment intensity rather than ICU admission, we aimed to evaluate an operational framework for risk stratification at MET activation.

## Methods

### Study design and data sources

This retrospective single-center cohort study was conducted to develop and internally validate prediction models for clinical outcomes following MET activation. The study included adult patients who experienced MET activation on general wards at Nippon Medical School Hospital, a tertiary-care academic hospital in Japan, between May 2021 and December 2025. The unit of analysis was the MET activation event. For patients with multiple activations during the same hospitalization, only the first activation was included to capture the initial episode of clinical deterioration and to avoid within-patient clustering.

Data were obtained from routinely collected electronic health records and the Diagnosis Procedure Combination (DPC) administrative claims database at Nippon Medical School Hospital [12]. Data for this study were accessed for research purposes between February 8, 2026 and March 2026. The authors did not have access to information that could identify individual participants during or after data collection.

The study protocol was approved by the Central Ethics Committee of Nippon Medical School (Approval No. M-2025–484). The approval was granted on February 7, 2026. The requirement for informed consent was waived because of the retrospective design and the use of anonymized clinical data.

This study was conducted and reported in accordance with the Transparent Reporting of a Multivariable Prediction Model for Individual Prognosis or Diagnosis (TRIPOD) statement.

### Patients and outcomes

Adult patients (aged ≥18 years) who experienced MET activation on general wards at Nippon Medical School Hospital during the study period were eligible for inclusion. When multiple MET activations occurred during the same hospitalization, only the first activation was analyzed to represent the initial episode of clinical deterioration. Patients were excluded if MET activation occurred on the day of hospital admission, if they were admitted to the coronary care unit (CCU) or stroke care unit (SCU) at the time of activation, or if a documented do-not-attempt-resuscitation (DNAR) order was present before or at the time of MET activation.

Two outcomes were prespecified and analyzed in parallel: (1) the initiation of ICU-level interventions within 24 hours after MET activation and (2) all-cause 14-day mortality. The 24-hour window for ICU-level intervention was calculated from the recorded time of MET activation in the electronic health record. ICU-level interventions were defined as the initiation of invasive mechanical ventilation, noninvasive positive-pressure ventilation, continuous vasopressor or inotropic infusion, renal replacement therapy, or polymyxin B hemoperfusion (PMX). These interventions were selected because they represent substantial escalation in treatment intensity, generally require close physiological monitoring or critical-care capability, and could be consistently identified from the available electronic clinical records. These interventions were defined independently of CCU or SCU admission in order to operationalize the need for intensive care based on treatment intensity rather than the location of care. Fourteen-day mortality was defined as death from any cause within 14 days after MET activation. Mortality status was determined using in-hospital clinical records and administrative data.

### Variables

All variables were defined using information available at or before the time of MET activation. Time-invariant baseline variables included age and sex. Cancer status was identified from the DPC administrative database and was defined as the presence of a diagnosis classified as a malignant neoplasm (ICD-10 codes C00–C97). Physiological variables were extracted from electronic health records and included heart rate, systolic blood pressure, and respiratory rate. Laboratory variables included serum albumin, blood urea nitrogen, C-reactive protein, potassium, and white blood cell count. For both vital signs and laboratory variables, measurements obtained from hospital admission to the time of MET activation were considered, and the measurement closest to MET activation was used. All candidate predictors were restricted to variables available at or before MET activation to ensure applicability for real-time clinical decision-making and triage.

### Model development

ICU-level intervention within 24 hours after MET activation and 14-day mortality were modeled using multivariable regression analyses. ICU-level intervention was analyzed using a logistic regression model, whereas 14-day mortality was analyzed using a Cox proportional hazards model with time from MET activation to death or censoring as the time scale. Candidate predictors were prespecified based on clinical relevance and availability at the time of MET activation. The study included all eligible MET activations during the prespecified study period, resulting in a fixed available sample size. Because the primary objective was prediction-model development rather than hypothesis testing, no conventional hypothesis-test-based power calculation was performed. Sample size was instead considered in relation to the number of outcome events and the number of predictor coefficients included in each primary model. The candidate predictor set was intentionally restricted in relation to the available number of outcome events (303 ICU-level intervention events and 147 deaths within 14 days) to mitigate the risk of overfitting. These variables included age, sex, serum albumin, blood urea nitrogen (BUN), C-reactive protein (CRP), potassium, white blood cell count, respiratory rate, heart rate, systolic blood pressure, and cancer status. Both primary models included 11 prespecified candidate predictors, each entered as a single linear term, yielding 11 predictor coefficients. This corresponded to approximately 27.5 ICU-level intervention events and 13.4 deaths per predictor coefficient for the ICU-level intervention and 14-day mortality models, respectively. In the main models, continuous predictors were entered as linear terms. To explore potential nonlinear associations, restricted cubic spline analyses for heart rate and systolic blood pressure were additionally performed as supplementary analyses, with four knots placed at the 5th, 35th, 65th, and 95th percentiles of their respective distributions. No missing values were observed in the variables used for model development and validation; therefore, all eligible patients were included in the analyses.

### Model performance and validation

Model discrimination was evaluated using the concordance index (C-index). Calibration was assessed using calibration intercepts and slopes derived from model recalibration, and calibration plots were constructed by comparing observed and predicted risks across deciles of predicted risk with loess smoothing. Internal validation was performed using bootstrap-based optimism correction. In 500 bootstrap samples, the models were refitted and evaluated in both the bootstrap samples and the original dataset. Optimism was calculated as the difference in performance between these evaluations, and optimism-corrected performance estimates were obtained by subtracting the mean optimism from the apparent performance. Ninety-five percent confidence intervals for the corrected metrics were estimated using the percentile method based on 2,000 bootstrap replicates.

Clinical utility was evaluated using decision curve analysis (DCA) [13]. Net benefit was calculated across a range of threshold probabilities and compared with treat-all and treat-none strategies. For graphical presentation of the joint predicted risks, thresholds of 0.30 for ICU-level intervention and 0.20 for 14-day mortality were used as illustrative visualization thresholds. These thresholds were not used for model development, internal validation, or decision curve analysis and should not be interpreted as clinically validated decision thresholds. For the two-dimensional visualization, predicted 14-day mortality probabilities were obtained from a supplementary logistic regression model restricted to patients with known 14-day outcome status. Known 14-day status was defined as death within 14 days or survival with at least 14 days of follow-up.

### Statistical analysis

Continuous variables are presented as medians with interquartile ranges, and categorical variables are presented as counts with percentages. Baseline characteristics were summarized descriptively without formal hypothesis testing because the primary objective of the study was prediction rather than causal inference. Baseline differences between groups were quantified using standardized mean differences (SMDs). To ensure reproducibility and enable individual risk estimation, the full regression coefficients of the logistic regression model, including the intercept, and those of the Cox proportional hazards model, along with baseline survival probabilities at selected time points, are provided in the Supplementary Materials. All statistical tests were two-sided, and p values <0.05 were considered statistically significant. All analyses were performed using R software (version 4.4.2). Logistic regression models were fitted using the stats package, Cox proportional hazards models using the survival package (version 3.7−0), and restricted cubic spline analyses using the rms package (version 7.0−0). Discrimination of the logistic regression model was assessed using the pROC package (version 1.18.5). Calibration, bootstrap-based internal validation, and decision curve analysis were implemented using custom R functions.

## Results

### Study population and baseline characteristics

During the study period, 852 adult patients with MET activation were screened. After applying the predefined exclusion criteria, 319 patients were excluded: MET activation on hospital day 1 (n = 176), CCU or SCU admission at or before MET activation (n = 73), and documented DNAR status at or before MET activation (n = 70) (Fig 1). The final analytic cohort consisted of 533 patients. Among these, 303 patients (56.8%) received ICU-level interventions within 24 hours after MET activation, whereas 230 patients (43.2%) did not. S1 Fig shows the distribution of time from hospital admission to MET activation. MET activities were more frequent during the early days of hospitalization and gradually decreased over time; however, events occurred throughout the entire hospitalization period, including beyond 60 days after admission.

**Fig 1:**
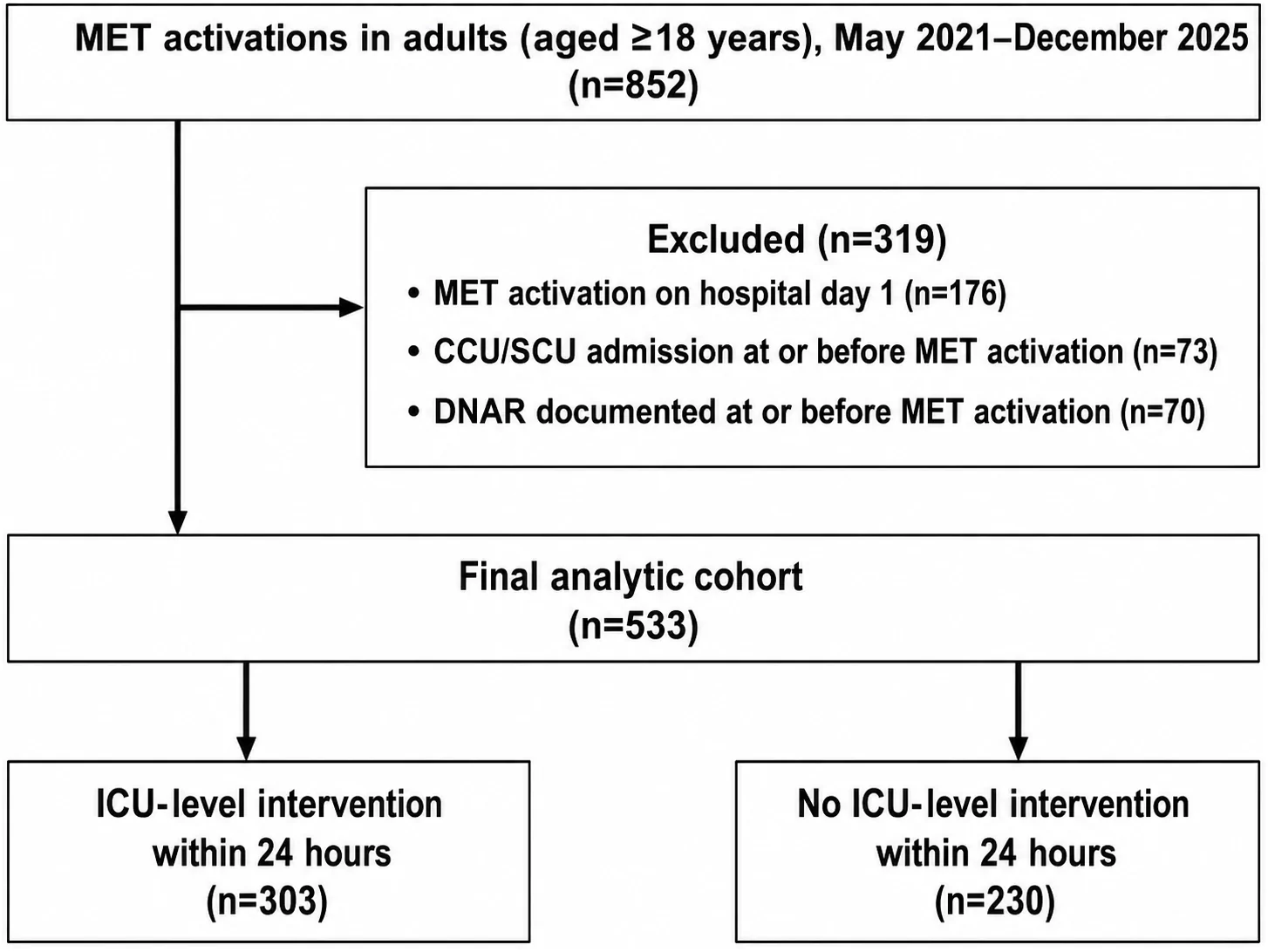
Study flow diagram of patient selection and outcome classification. Among 852 adult patients (aged ≥18 years) who experienced Medical Emergency Team (MET) activation between May 2021 and December 2025, 319 cases were excluded: MET activation on hospital day 1 (n = 176), admission to the coronary care unit (CCU) or stroke care unit (SCU) prior to or at the time of MET activation (n = 73), and documented do-not-attempt-resuscitation (DNAR) status at or before MET activation (n = 70). The final analytic cohort consisted of 533 patients. Within 24 hours after MET activation, 303 patients received ICU-level interventions, defined by the initiation of organ support, whereas 230 patients did not receive ICU-level interventions.

Baseline characteristics at the time of MET activation are summarized in Table 1. Compared with patients who did not require ICU-level interventions, those who required such interventions had a longer interval from admission to MET activation and a higher proportion of males, while age and emergency admission rates were similar between groups. At MET activation, the ICU-level intervention group demonstrated lower serum albumin levels and higher markers of renal dysfunction and systemic inflammation, including blood urea nitrogen, creatinine, C-reactive protein, total bilirubin, and white blood cell count. Hemoglobin levels were slightly lower in the ICU-level intervention group. Respiratory rate and heart rate were higher in the ICU-level intervention group, whereas systolic blood pressure and serum sodium levels were broadly comparable between groups.

**Table 1:** Baseline characteristics of patients with and without ICU-level intervention within 24 hours after MET activation.

Variable	Overall (n = 533)	ICU-level intervention (n = 303)	No ICU-level intervention (n = 230)	SMD
14-day mortality, n (%)	147 (27.6)	120 (39.6)	27 (11.7)	0.673
MET-to-event time, days	11 [5–26]	14 [6–30]	9 [4–22]	0.181
Age, years	73 [62–80]	72 [62–79]	74 [63–81]	0.020
Male, n (%)	341 (64.0)	207 (68.3)	134 (58.3)	0.210
Emergency admission, n (%)	302 (56.7)	172 (56.8)	130 (56.5)	0.005
Body mass index, kg/m²	21.2 [18.4–24.5]	21.3 [18.8–24.8]	21.0 [18.3–24.3]	0.167
Comorbidities				
Cancer, n (%)	259 (48.6)	157 (51.8)	102 (44.3)	0.150
Cardiac disease, n (%)	136 (25.5)	82 (27.1)	54 (23.5)	0.083
Neurologic disease, n (%)	122 (22.9)	68 (22.4)	54 (23.5)	0.025
Trauma, n (%)	46 (8.6)	29 (9.6)	17 (7.4)	0.078
Laboratory data				
Albumin, g/dL	2.6 [2.0–3.1]	2.4 [1.9–2.9]	2.9 [2.4–3.3]	0.646
Blood urea nitrogen, mg/dL	22.2 [14.5–38.8]	27.5 [17.1–44.6]	18.2 [12.4–29.0]	0.515
C-reactive protein, mg/dL	4.34 [0.92–11.78]	6.39 [1.54–14.86]	2.54 [0.52–7.56]	0.512
Creatinine, mg/dL	1.01 [0.68–1.69]	1.17 [0.72–2.08]	0.85 [0.65–1.30]	0.178
Hemoglobin, g/dL	9.8 [8.3–11.6]	9.5 [8.2–11.3]	10.2 [8.7–11.7]	0.216
Potassium, mmol/L	4.10 [3.60–4.70]	4.30 [3.70–5.00]	4.00 [3.50–4.40]	0.443
Sodium, mmol/L	138 [135–141]	138 [134–142]	138 [135–141]	0.108
Total bilirubin, mg/dL	0.58 [0.36–0.95]	0.64 [0.38–1.06]	0.51 [0.34–0.82]	0.171
White blood cells, × 10³/µL	9.7 [5.8–14.5]	11.1 [6.0–16.8]	8.5 [5.8–12.7]	0.258
Vital signs				
Body temperature, °C	36.7 [36.4–37.0]	36.7 [36.5–37.0]	36.7 [36.4–37.1]	0.014
Respiratory rate,/min	17 [15 –18]	17 [15 –18]	16 [15 –18]	0.125
Heart rate,/min	84 [73–99]	86 [74–102]	82 [71–96]	0.220
Systolic blood pressure, mmHg	126 [111–141]	126 [110–142]	126 [112–140]	0.033

**Footnote**

Values are presented as median [interquartile range] for continuous variables and number (percentage) for categorical variables.

Standardized mean differences (SMDs) were calculated to assess the magnitude of differences between the ICU-level intervention and no ICU-level intervention groups.

**Abbreviations**

MET, medical emergency team; SMD, standardized mean difference.

The distribution of clinical outcomes is presented in S1 Table. Fourteen-day mortality occurred in 147 patients (27.6%). Within 24 hours after MET activation, 303 patients (56.8%) required ICU-level interventions. Vasopressor or inotropic infusion was administered to 262 patients (49.2%), mechanical ventilation was initiated in 206 patients (38.6%), and renal replacement therapy or PMX was initiated in 47 patients (8.8%).

### Multivariable Logistic Regression Analysis

In the multivariable logistic regression analysis, lower serum albumin levels and higher levels of BUN, CRP, potassium, and white blood cell count were significantly associated with ICU-level intervention within 24 hours after MET activation (Table 2). Serum albumin showed an inverse association with ICU-level intervention (OR 0.527, 95% CI 0.392–0.707, p < 0.001). In contrast, higher BUN (OR 1.018, 95% CI 1.006–1.029, p = 0.002), CRP (OR 1.029, 95% CI 1.003–1.056, p = 0.030), potassium (OR 1.296, 95% CI 1.054–1.594, p = 0.014), and white blood cell count (OR 1.030, 95% CI 1.004–1.057, p = 0.026) were associated with increased odds of ICU-level intervention. Age, sex, respiratory rate, heart rate, systolic blood pressure, and cancer status were not significantly associated with ICU-level intervention.

**Table 2:** Multivariable Logistic Regression Analysis for ICU-Level Intervention.

Variable	Odds Ratio (95% CI)	P value
Age	0.987 (0.974–1.001)	0.074
Male	1.303 (0.871–1.951)	0.198
Albumin	0.527 (0.392–0.707)	<0.001
Blood urea nitrogen	1.018 (1.006–1.029)	0.002
C-reactive protein	1.029 (1.003–1.056)	0.03
Potassium	1.296 (1.054–1.594)	0.014
White blood cells	1.030 (1.004–1.057)	0.026
Respiratory rate	1.015 (0.975–1.057)	0.468
Heart rate	1.007 (0.996–1.017)	0.2
Systolic blood pressure	1.000 (0.993–1.007)	0.988
Cancer	1.182 (0.798–1.753)	0.404

Odds ratios (ORs) are presented for interpretability; full regression coefficients are provided in the S2 Table.

Full regression coefficients, including the model intercept, are provided in S2 Table.

In the Cox proportional hazards analysis for 14-day mortality (Table 3), lower serum albumin levels and higher levels of BUN, potassium, white blood cell count, and heart rate were significantly associated with increased mortality risk. Serum albumin was inversely associated with mortality (HR 0.626, 95% CI 0.484–0.810, p < 0.001). Higher BUN (HR 1.016, 95% CI 1.010–1.023, p < 0.001), potassium (HR 1.474, 95% CI 1.333–1.629, p < 0.001), white blood cell count (HR 1.006, 95% CI 1.002–1.011, p = 0.007), and heart rate (HR 1.011, 95% CI 1.003–1.020, p = 0.009) were associated with higher mortality risk. Age, sex, CRP, respiratory rate, systolic blood pressure, and cancer status were not significantly associated with 14-day mortality. Full regression coefficients and baseline survival probabilities are provided in S3 and S4 Tables to enable individual risk estimation.

**Table 3:** Multivariable cox proportional hazards model for 14-day mortality.

Variable	Hazard Ratio (95% CI)	P value
Age	1.004 (0.992–1.017)	0.502
Male	0.952 (0.669–1.354)	0.783
Albumin	0.626 (0.484–0.810)	<0.001
Blood urea nitrogen	1.016 (1.010–1.023)	<0.001
C-reactive protein	1.005 (0.987–1.025)	0.576
Potassium	1.474 (1.333–1.629)	<0.001
White blood cells	1.006 (1.002–1.011)	0.007
Respiratory rate	1.016 (0.985–1.048)	0.324
Heart rate	1.011 (1.003–1.020)	0.009
Systolic blood pressure	0.998 (0.992–1.005)	0.594
Cancer	1.180 (0.833–1.671)	0.352

Hazard ratios (HRs) are presented; full regression coefficients and baseline survival probabilities are provided in the Supplementary S3 and S4 Tables.

The nonlinear associations of heart rate and systolic blood pressure with ICU-level intervention and 14-day mortality were further explored using restricted cubic spline models (S2 and S3 Figs). For ICU-level intervention, restricted cubic spline analysis did not demonstrate a clear association between heart rate or systolic blood pressure and the outcome, with relatively flat risk patterns across the observed ranges (S2 Fig). For 14-day mortality, the spline curve suggested a gradual increase in relative hazard at higher heart rate levels, although the overall association was not statistically significant (S3 Fig).

### Model performance

The predictive performance of the models is shown in Fig 2 and S4 and S5 Figs. The model for ICU-level intervention within 24 hours demonstrated moderate discrimination, with a C-index of 0.721 (95% CI 0.660–0.745) (Fig 2A). The supplementary logistic regression model for 14-day mortality among patients with known 14-day outcome status showed good discrimination, with a C-index of 0.784 (95% CI 0.720–0.814) (Figure 2B).

**Fig 2:**
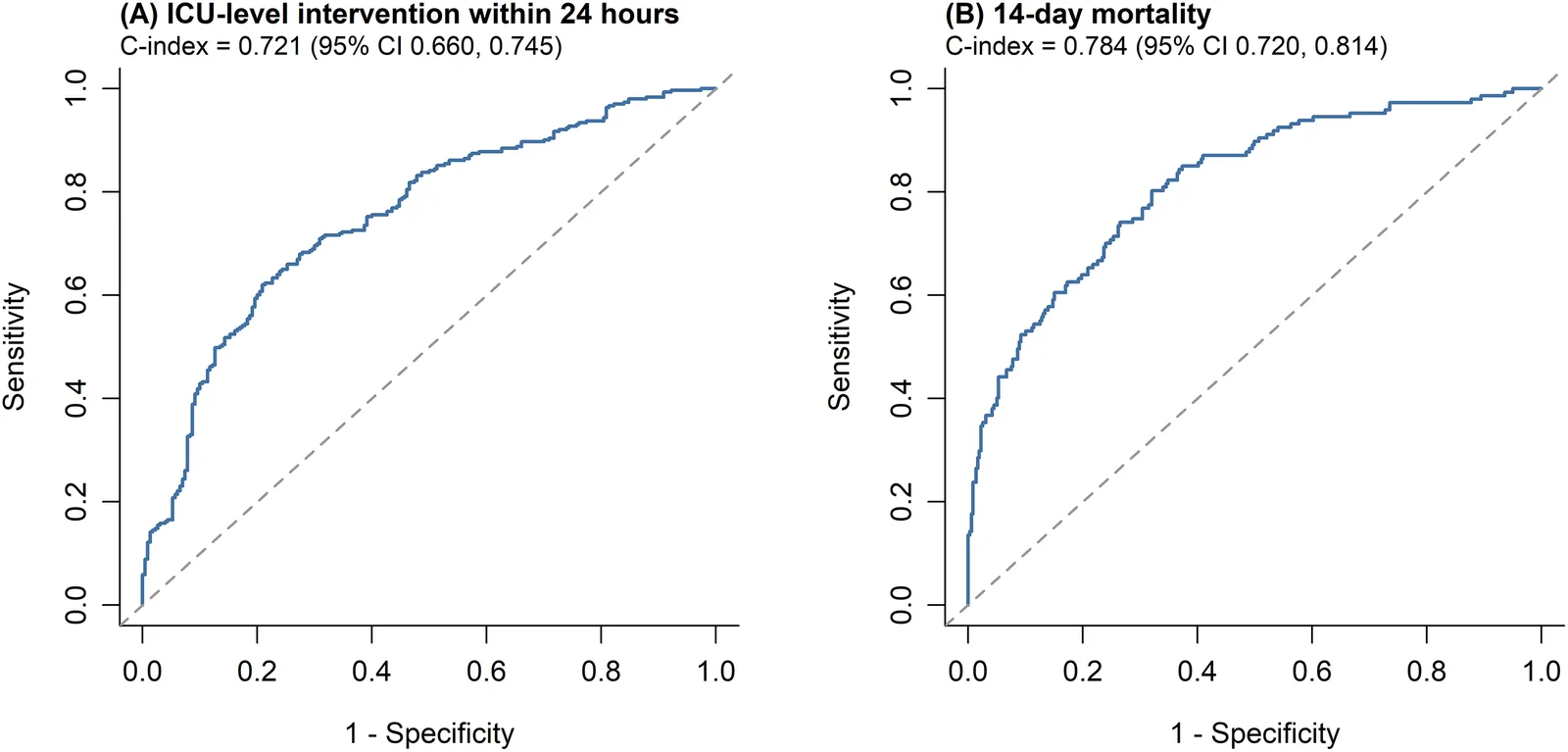
Discrimination performance of the prediction models. (A) Receiver operating characteristic (ROC) curve for the prediction of ICU-level intervention within 24 hours after MET activation. The model demonstrated moderate discrimination with a C-index of 0.721 (95% CI 0.660–0.745). (B) Receiver operating characteristic (ROC) curve for the supplementary logistic regression model predicting 14-day mortality among patients with known 14-day outcome status. The model showed good discrimination with a C-index of 0.784 (95% CI 0.720–0.814). The dashed diagonal line represents the line of no discrimination.

Calibration plots indicated generally good agreement between predicted and observed risks for both outcomes (S4 Fig). The optimism-corrected calibration intercept and slope were 0.033 (95% CI −0.273 to 0.401) and 0.821 (95% CI 0.299 to 1.019), respectively, for ICU-level intervention, and −0.127 (95% CI −0.708 to 0.220) and 0.834 (95% CI 0.318 to 1.041), respectively, for 14-day mortality.

Decision curve analysis demonstrated that the prediction models provided greater net benefit than the treat-all or treat-none strategies across clinically relevant threshold probabilities (S5 Fig). The model for ICU-level intervention showed net benefit particularly within threshold probabilities of approximately 0.10–0.45, whereas the 14-day mortality model demonstrated consistent clinical utility across a broad range of threshold probabilities.

### Clinical risk stratification

Clinical risk stratification based on the joint distribution of predicted probabilities for ICU-level intervention and 14-day mortality is shown in Fig 3. This exploratory analysis included 506 patients with known 14-day outcome status. Using illustrative thresholds of 0.30 for ICU-level intervention and 0.20 for 14-day mortality, patients were classified into risk regions for graphical presentation. Region A included patients with a low predicted risk of 14-day mortality but a high predicted probability of ICU-level intervention (n = 186). In this group, the observed rate of ICU-level intervention was 46.8%, whereas the 14-day mortality rate was relatively low at 11.8%. Region B consisted of patients with both high predicted ICU intervention probability and high predicted mortality risk (n = 252). This group demonstrated the highest observed event rates, with ICU-level intervention occurring in 74.2% of patients and 14-day mortality in 48.0%. Region C represented patients with low predicted probabilities for both ICU-level intervention and mortality (n = 68). In this group, ICU-level intervention occurred in 27.9% of patients and 14-day mortality in 5.9%.

**Fig 3:**
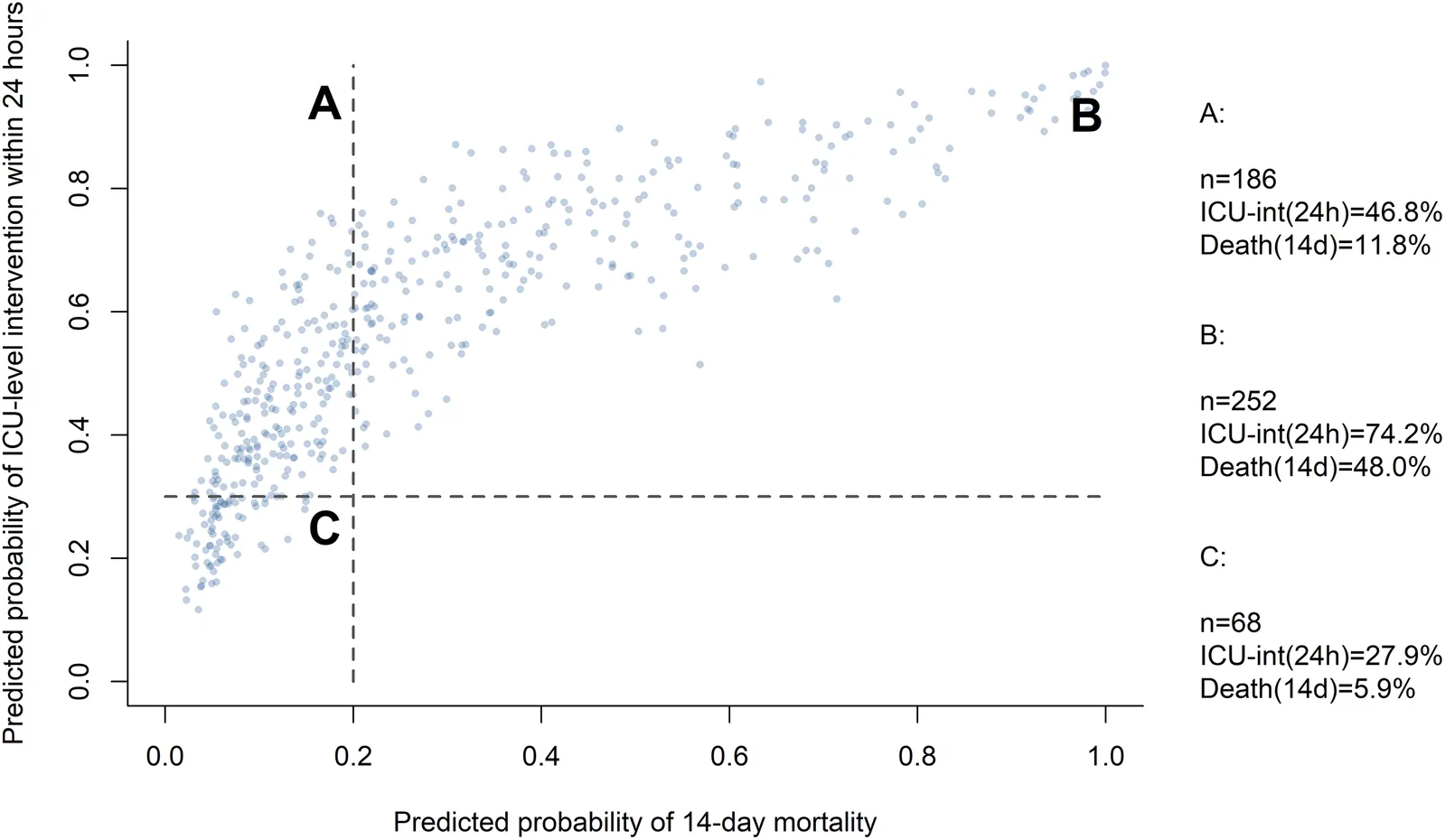
Integrated risk stratification based on predicted probabilities of ICU-level intervention and 14-day mortality. The x-axis indicates the predicted probability of 14-day mortality, and the y-axis indicates the predicted probability of ICU-level intervention within 24 hours after MET activation. Each point represents a patient with known 14-day outcome status (n = 506). The predicted probability of 14-day mortality was derived from the supplementary logistic regression model. Dashed lines indicate the illustrative thresholds of 0.20 for predicted 14-day mortality and 0.30 for predicted ICU-level intervention, which were selected post hoc for visualization purposes and do not represent validated clinical decision thresholds. Group A included patients with higher predicted probability of ICU-level intervention but lower predicted probability of 14-day mortality (n = 186; ICU-level intervention within 24 hours: 46.8%; 14-day mortality: 11.8%). Group B included patients with high predicted probabilities for both ICU-level intervention and 14-day mortality (n = 252; ICU-level intervention within 24 hours: 74.2%; 14-day mortality: 48.0%). Group C included patients with lower predicted probabilities for both outcomes (n = 68; ICU-level intervention within 24 hours: 27.9%; 14-day mortality: 5.9%). This two-dimensional framework illustrates the heterogeneity of clinical trajectories after MET activation and allows simultaneous assessment of treatment intensity and short-term mortality risk.

## Discussion

In this retrospective cohort study of patients undergoing MET activation on general wards, we developed and internally validated prediction models for ICU-level intervention within 24 hours and 14-day mortality using routinely available clinical variables. Beyond model performance, the findings highlight that MET activation represents a critical transition point at which two related but distinct processes occur: escalation of treatment intensity and short-term mortality risk. By defining intensive care need according to organ support rather than ICU admission, this study separated treatment intensity from location of care and demonstrated that these dimensions, although correlated, are not interchangeable. The differing patterns of associated risk factors suggest that escalation decisions and mortality risk may reflect partially distinct pathophysiological and clinical processes. These findings extend existing MET prediction research by evaluating treatment escalation according to treatment intensity rather than ICU admission, while assessing short-term mortality in parallel.

Previous post-MET prediction studies, including that of Batterbury et al., have evaluated outcomes such as unplanned ICU admission and mortality [9]. The present study differs by defining treatment escalation according to the initiation of ICU-level interventions irrespective of the location of care. Reported discrimination has generally ranged from approximately 0.70 to 0.85, with mortality models often achieving AUC values around 0.75 and ICU transfer models typically ranging from 0.65 to 0.75 [14–16]. However, many of these studies have relied on ICU admission as a surrogate for intensive care needs [17, 18], and relatively few have evaluated the initiation of organ support directly. In the present study, discrimination was comparable to prior reports, with a C-index of 0.721 for ICU-level intervention and 0.784 for 14-day mortality. Importantly, discrimination above 0.70 was observed even when treatment intensity, rather than ICU admission, was used to define the outcome [19]. In addition, model calibration and bootstrap-based internal validation were performed, providing a comprehensive evaluation of model performance beyond discrimination alone [20]. By evaluating ICU-level intervention and mortality in parallel, the present approach provides a broader characterization of clinical risk at the time of MET activation.

Risk factors associated with ICU-level intervention and mortality partially overlapped but were not identical. Hypoalbuminemia, elevated blood urea nitrogen, and leukocytosis were associated with both outcomes, consistent with evidence that impaired physiological reserve and systemic inflammation contribute to clinical deterioration and short-term mortality [21–23]. Higher potassium levels were associated with both ICU-level intervention and 14-day mortality, with a stronger association observed for mortality, suggesting that disturbances in potassium homeostasis may be particularly relevant to short-term mortality risk [24]. Heart rate was not independently associated with ICU-level intervention, whereas supplementary spline analyses suggested a possible nonlinear association with mortality, potentially reflecting sympathetic activation and physiological stress affecting survival rather than escalation decisions [25]. These findings suggest that treatment intensity and mortality represent related but partially distinct clinical constructs and should not be considered interchangeable outcomes in prediction modeling.

The two-dimensional risk framework proposed in this study may support clinical decision-making at the time of MET activation. By jointly estimating the probabilities of ICU-level intervention and short-term mortality, the model provides a multidimensional representation of patient risk that extends beyond conventional single-outcome prediction [25, 26]. Although illustrative thresholds were used to aid visualization, the predicted risks themselves were continuous (Fig 3). The thresholds were selected solely for visualization and should not be interpreted as clinically actionable cutoffs; determination of such thresholds would require external validation and evaluation of the consequences of different decision thresholds. Because the model relies exclusively on routinely available vital signs and laboratory variables measured at the time of MET activation, it may be feasible to integrate this approach into electronic health record systems with minimal additional data burden [25–27]. Visualizing predicted probabilities within a two-axis framework may also facilitate shared situational awareness and interdisciplinary communication during decisions regarding escalation of care. However, this study was conducted at a single center and included internal validation only. External validation is required to assess the generalizability and transportability of the model across different clinical settings. In addition, prospective studies are needed to determine whether implementation of this prediction framework improves clinical decision-making or patient-centered outcomes. Therefore, the model should be regarded as a decision-support tool to assist risk communication and clinical deliberation rather than an autonomous triage system.

Several limitations should be acknowledged. First, this study was conducted at a single tertiary-care center, which may limit generalizability to other settings with different patient populations, MET structures, and escalation practices. In addition, our MET system includes both rapid response system activations and code blue events, potentially resulting in a cohort with greater illness severity than in studies restricted to early deterioration. This difference in case mix should be considered when comparing our findings with prior MET or RRS studies. Second, although predictors were defined using information available at or before MET activation, some laboratory variables may not be immediately available in real time. Therefore, the models may reflect risk stratification at the time of MET evaluation rather than early prediction before clinical deterioration. This distinction suggests that the model is more appropriately interpreted as a decision-support tool at MET activation rather than an early warning system. Third, no missing values were observed in the variables used for model development. In addition, the initiation of organ support may be influenced by institutional practices and clinician judgment, which could affect the generalizability of the outcome definition. Finally, only internal validation was performed. External validation and prospective studies are required to assess the transportability and clinical utility of this framework.

## Conclusion

In patients undergoing MET activation, prediction models using routinely available clinical variables demonstrated moderate-to-good discrimination for ICU-level intervention within 24 hours and 14-day mortality. Defining escalation of care based on treatment intensity rather than ICU admission may provide an operational framework for distinguishing treatment intensity from the location of care. However, given the single-center design, the generalizability of this approach remains uncertain, and external validation is required before clinical implementation.

### AI Disclosure

Artificial intelligence–assisted technology was used to support programming and code refinement for statistical analyses. All analytical procedures, results, and interpretations were independently verified by the authors, who take full responsibility for the integrity and accuracy of the manuscript.

## Supporting information

S1 Table ICU-level interventions and outcomes. (DOCX)

S2 Table Full model coefficients for the logistic regression model predicting ICU-level intervention within 24 hours after MET activation. (DOCX)

S3 Table Full model coefficients for the Cox proportional hazards model predicting 14-day mortality. (DOCX)

S4 Table Baseline survival probabilities for the Cox proportional hazards model. (DOCX)

S1 Fig Distribution of time from hospital admission to MET activation. Histogram showing the distribution of days from hospital admission to medical emergency team (MET) activation among the study population. The majority of MET activations occurred during the early phase of hospitalization, whereas a smaller proportion occurred later during the hospital stay. The final category (≥60 days) represents patients whose MET activation occurred 60 days or more after admission.
(TIFF)

S2 Fig Nonlinear associations between vital signs and ICU-level intervention. Restricted cubic spline curves showing the associations of (A) heart rate and (B) systolic blood pressure (SBP) with the odds of ICU-level intervention within 24 hours after MET activation. Solid lines represent the estimated relative odds, and dashed lines represent the 95% confidence intervals. The reference value was set at the median of each variable. The horizontal dotted line represents the reference level (odds ratio = 1.0).
(TIFF)

S3 Fig Nonlinear associations between vital signs and 14-day mortality. Restricted cubic spline curves showing the associations of (A) heart rate and (B) systolic blood pressure (SBP) with the hazard of 14-day mortality after MET activation. Solid lines represent the estimated relative hazard, and dashed lines represent the 95% confidence intervals. The reference value was set at the median of each variable. The horizontal dotted line represents the reference level (hazard ratio = 1.0).
(TIFF)

S4 Fig Calibration plots of the prediction models. (A) Calibration plot for the prediction model of ICU-level intervention within 24 hours after MET activation. (B) Calibration plot for the prediction model of 14-day mortality in the sensitivity analysis. The dashed line represents ideal calibration, and the solid line represents the bootstrap bias-corrected calibration curve. Points indicate observed event rates across grouped predicted probabilities. The optimism-corrected calibration intercept and slope were 0.033 and 0.821 for the ICU-level intervention model and −0.127 and 0.834 for the 14-day mortality model, respectively.
(TIFF)

S5 Fig Decision curve analysis of the prediction models. (A) Decision curve for the prediction model of ICU-level intervention within 24 hours after MET activation. (B) Decision curve for the prediction model of 14-day mortality in the sensitivity analysis. The x-axis represents the threshold probability, and the y-axis represents the net benefit. The solid line represents the prediction model, the dashed line represents the treat-all strategy, and the dotted line represents the treat-none strategy.
(TIFF)
