# Supplementary material for: Development and internal validation of prediction models for ICU-level intervention and 14-day mortality at Medical Emergency Team activation: A retrospective cohort study
Source: PLoS One. 2026 Oct 9;21(10):e0360511. doi: 10.1371/journal.pone.0360511 (PMC13652246; doi:10.1371/journal.pone.0360511)
Supplement: Supplementary file 1 [file pone.0360511.s001.docx]

**S1 Table. ICU-Level Interventions and Outcomes**

| **Variable** | **n (%)** |
| --- | --- |
| 14-day mortality | 147 (27.6) |
| ICU-level intervention | 303 (56.8) |
| Renal replacement therapy or PMX | 47 (8.8) |
| Catecholamine administration | 262 (49.2) |
| Mechanical ventilation | 206 (38.6) |

**Footnote**

Values are presented as number (percentage) of the total study population (n=533). ICU-level intervention was defined as the initiation within 24 hours after Medical Emergency Team activation of invasive or noninvasive mechanical ventilation, continuous vasopressor or inotropic infusion, renal replacement therapy, or polymyxin B hemoperfusion. Individual ICU-level interventions were not mutually exclusive; therefore, their counts and percentages do not sum to the total number of patients receiving ICU-level interventions. PMX, polymyxin B hemoperfusion.
