# Supplementary material for: Development and internal validation of prediction models for ICU-level intervention and 14-day mortality at Medical Emergency Team activation: A retrospective cohort study
Source: PLoS One. 2026 Oct 9;21(10):e0360511. doi: 10.1371/journal.pone.0360511 (PMC13652246; doi:10.1371/journal.pone.0360511)
Supplement: Supplementary file 2 [file pone.0360511.s002.docx]

**S2 Table Full model coefficients for the logistic regression model predicting ICU-level intervention within 24 hours**

| **Term** | **β** | **SE** |
| --- | --- | --- |
| Intercept | -0.385 | 1.058 |
| Age | -0.013 | 0.007 |
| Male | 0.265 | 0.206 |
| Albumin | -0.641 | 0.15 |
| Blood urea nitrogen | 0.017 | 0.006 |
| C-reactive protein | 0.029 | 0.013 |
| Potassium | 0.259 | 0.106 |
| White blood cells | 0.029 | 0.013 |
| Respiratory rate | 0.015 | 0.021 |
| Heart rate | 0.007 | 0.005 |
| Systolic blood pressure | 0.00006 | 0.004 |
| Cancer | 0.168 | 0.201 |
