# Supplementary material for: Development and internal validation of prediction models for ICU-level intervention and 14-day mortality at Medical Emergency Team activation: A retrospective cohort study
Source: PLoS One. 2026 Oct 9;21(10):e0360511. doi: 10.1371/journal.pone.0360511 (PMC13652246; doi:10.1371/journal.pone.0360511)
Supplement: Supplementary file 3 [file pone.0360511.s003.docx]

**S3 Table Full model coefficients for the Cox proportional hazards model predicting 14-day mortality**

| Term | β | SE |
| --- | --- | --- |
| Age | 0.004 | 0.007 |
| Male | -0.05 | 0.18 |
| Albumin | -0.468 | 0.131 |
| Blood urea nitrogen | 0.016 | 0.003 |
| C-reactive protein | 0.005 | 0.01 |
| Potassium | 0.388 | 0.051 |
| White blood cells | 0.006 | 0.002 |
| Respiratory rate | 0.016 | 0.016 |
| Heart rate | 0.011 | 0.004 |
| Systolic blood pressure | -0.002 | 0.003 |
| Cancer | 0.165 | 0.178 |
