# Supplementary material for: Development and internal validation of prediction models for ICU-level intervention and 14-day mortality at Medical Emergency Team activation: A retrospective cohort study
Source: PLoS One. 2026 Oct 9;21(10):e0360511. doi: 10.1371/journal.pone.0360511 (PMC13652246; doi:10.1371/journal.pone.0360511)
Supplement: Supplementary file 4 [file pone.0360511.s004.docx]

**S4 Table Baseline survival probabilities for the Cox proportional hazards model**

| **Time (days)** | **Baseline survival** |
| --- | --- |
| 1 | 0.914 |
| 3 | 0.871 |
| 5 | 0.836 |
| 7 | 0.826 |
| 10 | 0.797 |
| 14 | 0.778 |
